# JSI124 inhibits breast cancer cell growth by suppressing the function of B cells via the downregulation of signal transducer and activator of transcription 3

**DOI:** 10.3892/ol.2014.2221

**Published:** 2014-06-04

**Authors:** YI REN, KUN YU, SU’AN SUN, ZHI LI, JIN YUAN, XUE DONG HAN, JIANHUA SHI, LINLIN ZHEN

**Affiliations:** 1 Department of Breast and Thyroid Surgery, Huai’an First People’s Hospital, Huai’an, Jiangsu 223001, P.R. China; 2 Department of Cardiology, Huai’an First People’s Hospital, Huai’an, Jiangsu 223001, P.R. China; 3 Department of Pathology, Huai’an First People’s Hospital, Huai’an, Jiangsu 223001, P.R. China

**Keywords:** JSI124, 4T1 tumor, B cells, signal transducer and activator of transcription 3

## Abstract

JSI-124, also known as cucurbitacin I, is a selective inhibitor of Janus kinase/signal transducer and activator of transcription 3 (JAK/STAT3), and *in vitro* and *in vivo* studies have found that it has anti-tumor and anti-proliferative properties. However, the role of JSI124 in tumor-associated B cells has yet to be elucidated. The present study demonstrated that STAT3 is significantly activated in the B cells of patients with breast cancer. Furthermore, a 4T1 tumor-bearing mouse model revealed that JSI124 effectively inhibited tumor growth. Moreover, the STAT3 levels in the B cells of the JSI124-treated mice were found to be significantly decreased. B cells from normal Balb/c mice, the 4T1-bearing mice and the JSI124-treated 4T1 mice were purified and intravenously injected into the 4T1-bearing Balb/c mice. Tumor growth data showed that the 4T1 tumor mouse-derived B cells, which exhibited a higher level of STAT3, promoted tumor growth, while the JSI124-treated 4T1 mouse-derived B cells had a tumor suppressor function. Furthermore, the B cells from the normal Balb/c mice were treated with phosphate-buffered saline, JSI124 and 4T1 tumor cells, then the B cell STAT3 levels were analyzed. Following injection into the 4T1 mice, the 4T1 cell-treated B cells were observed to enhance tumor growth, while the JSI124-treated B cells were found to inhibit the growth of 4T1 tumors *in vivo*. These findings show a novel role of JSI124 in tumor suppression through the downregulation of the expression of STAT3 in tumor-associated B cells.

## Introduction

JSI-124 is a potent inhibitor of the signal transducer and activator of transcription 3 (STAT3) signaling pathway (1). Previous studies have reported that JSI124 has anti-tumor activities in human breast cancer (2), lung cancer (3), neuroblastoma (4,5), murine melanoma cell lines (6) and B-cell leukemia (7). The mechanisms underlying this anti-tumor activity include the activation of the nuclear factor κ-light-chain-enhancer of activated B cells pathway in human glioblastoma cells (8), Rac 1 inhibition in breast cancer cells by a reactive oxygen species-mediated function (9), diminishing self-renewing and radiochemoresistant abilities in thyroid cancer-derived cluster of differentiation (CD)133^+^ cells (10) and the suppression of cell motility through indirectly interfering with actin dynamics in B16-F1 mouse melanoma cells (11). Su *et al* (12) also reported that the G_2_/M-phase cell cycle arrest and apoptosis augmentation caused by JSI124 inhibits glioblastoma multiforme cell proliferation.

B cells are prevalent in various tumor types and are found primarily at inflammatory sites in aggregates with other immune cells. Yang *et al* (13) reported that B cells may provide a contribution to a network with other cells in order to promote STAT3-dependent tumor angiogenesis. Consistent with this, STAT3 has been shown to be important in the regulation of the multi-directional feed-forward loop between tumor-associated myeloid cells, endothelial cells and tumor cells in tumor angiogenesis (14). However the association between JSI124 and STAT3 levels in B cells has yet to be elucidated.

In this study, the expression of STAT3 in the B cells of breast cancer patients was first detected and a mouse 4T1 breast cancer model was further applied for revealing a novel mechanism of tumor suppression by JSI124.

## Materials and methods

### Cell culture and mice

4T1 mouse breast tumor cells were purchased from the American Type Culture Collection (Manassas, VA, USA) and cultured in Dulbecco’s modified Eagle’s medium supplemented with 10% fetal calf serum, 100 U/ml penicillin and 100 μg/ml streptomycin in a humidified incubator at 37°C with 5% CO_2_.

BALB/c mice were purchased from the Experimental Animal Center of Nanjing Medical University (Nanjing, China) and maintained under pathogen-free conditions according to protocols that were approved by the Jiangsu Province Animal Care and Use Committee.

### Human blood samples

Peripheral blood samples were obtained from nine healthy individuals and 10 patients with breast cancer between 2011 and 2013. All the blood samples were collected subsequent to obtaining written informed consent according to a protocol approved by the Institutional Review Board of the First People’s Hospital of Huai’an (Huai’an, China).

### Western blot analysis

In brief, B cells that were purified from human blood or mouse spleens were lysed and the proteins of the lysed cells were separated on 12% polyacrylamide gels using SDS-PAGE. The separated proteins were transferred onto nitrocellulose membranes and western blot analysis was performed using phospho-Stat3 (Tyr705) (3E2) rabbit anti-mouse monoclonal antibody (Cell Signaling Technology, Inc., Beverly, MA, USA) and a rabbit anti-mouse β-actin polyclonal antibody as the control (Santa Cruz Biotechnology, Inc., Santa Cruz, CA, USA).

### B-cell isolation

To prepare the B cells from the human blood samples, the peripheral blood from nine healthy volunteers and 10 patients with breast cancer was collected. CD19^+^ B cells from the whole blood were isolated using Dynabeads^®^ CD19 (Invitrogen Life Technologies, Carlsbad, CA, USA), according to the manufacturer’s instructions.

In order to isolate the B cells from the mouse spleens, splenocytes were prepared using homogenization. The red blood cells were removed through lysis using ammonium-chloride-potassium buffer, and following two washes in pre-iced phosphate-buffered saline (PBS), the CD19^+^ B cells were purified using Dynabeads CD19 (Invitrogen Life Technologies).

### In vivo tumor experiments

To confirm the anti-4T1 tumor function of JSI124 *in vivo*, 2×10^5^ 4T1 cells were subcutaneously injected into the Balb/c mice. Tumor growth was assessed every five days and the expression of STAT3 was analyzed in the B cells (1×10^6^) from the PBS- and JSI124-treated mice using western blot analysis.

To investigate the effect of B cells on 4T1 tumor growth, 4T1-bearing Balb/c mice were treated with normal Balb/c mouse-derived B cells, 4T1-bearing mouse-derived B cells or JSI124-treated 4T1-bearing mouse-derived B cells (2×10^5^ cells each time) every three days, for five times. Tumor growth was determined by measuring the tumor volume.

To further investigate the function of JSI124-treated B cells on tumor growth, B cells were treated with JSI124 or incubated with 4T1 cells *in vitro* for 24 h, then the B cells were intravenously injected into 4T1-implanted mice and the tumor volume was measured every five days.

## Results

### STAT3 expression is increased in B cells from the peripheral blood of patients with breast cancer

**Figure 1:**
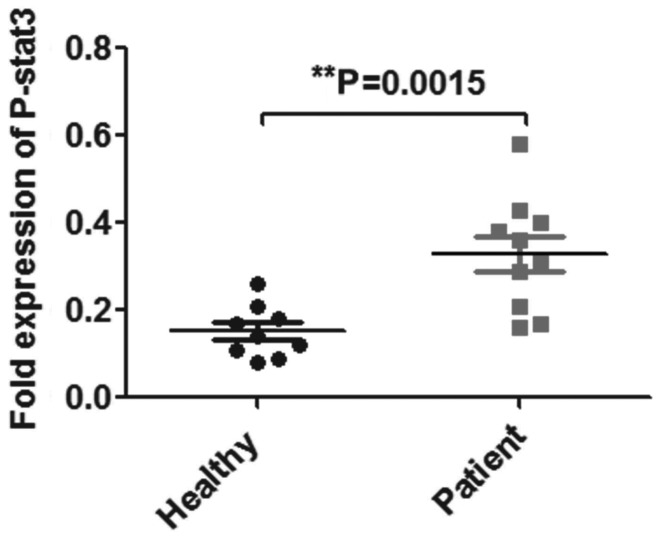
Expression of STAT3 in healthy individuals and patients with breast cancer. STAT3 expression was detected in 9 healthy individuals and 10 patients with breast cancer. P-STAT3, phosphorylated signal transducer and activator of transcription 3.

STAT3 expression was assessed in the B cells from the peripheral blood of nine healthy individuals and 10 patients with breast cancer. As shown in Fig. 1, STAT3 expression was upregulated approximately two-fold in the B cells from the patients with breast cancer compared with those in the healthy individuals (P=0.0015).

### JSI124 suppresses 4T1 tumor growth through the inhibition of STAT3 expression in B cells

**Figure 2:**
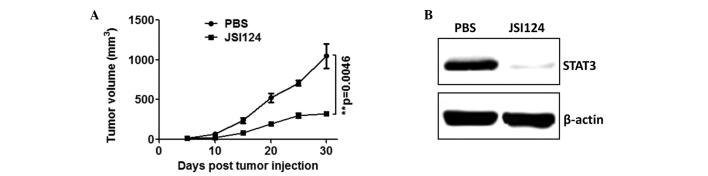
JSI124 inhibits 4T1 tumor growth and STAT3 expression. (A) 4T1 tumor volume in mice treated with JSI124 or PBS. (B) STAT3 expression in the B cells of 4T1-bearing mice treated with PBS and JSI124. ^**^P=0.0046. STAT3, signal transducer and activator of transcription 3; PBS, phosphate-buffered saline.

JSI124 was used to treat the 4T1 tumor-bearing mice and tumor growth was measured every five days. As shown in Fig. 2A, JSI124 was found to significantly inhibit 4T1 tumor growth (P=0.0046). The expression of STAT3 in the B cells of the JSI124- and PBS-treated mice was assessed using western blot analysis. As shown in Fig. 2B, STAT3 expression was observed to be markedly downregulated in the B cells of the 4T1-bearing mice treated with JSI124 compared with those treated with PBS.

### JSI124-treated 4T1 mouse-derived B cells suppress 4T1 tumor growth

**Figure 3:**
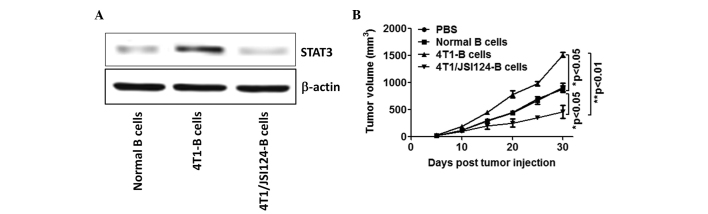
STAT3 expression in B cells and tumor growth following injection with B cells from tumor-bearing mice with or without JSI124 treatment. (A) STAT3 expression in B cells from normal mice, 4T1-bearing mice and JSI124-treated 4T1 tumor-bearing mice, detected using western blot analysis. (B) 4T1 tumor volume was measured following treatment with B cells from normal mice, 4T1-bearing mice and JSI124-treated 4T1-bearing tumor mice. ^*^P<0.05; ^**^P<0.01. STAT, signal transducer and activator of transcription; PBS, phosphate-buffered saline.

Previous studies have reported that STAT3 levels in B cells are associated with tumor growth (13), therefore, the present study investigated the effect of JSI124-treated 4T1 mouse-derived B cells on 4T1 tumor growth *in vivo*. As shown in Fig. 3A, the expression of STAT3 in the JSI124-treated 4T1 mouse-derived B cells was significantly downregulated compared with the B cells from the 4T1-bearing mice. *In vivo* tumor volume data revealed that the B cells from the 4T1-bearing mice promoted tumor growth (P<0.05); however, the JSI124-treated 4T1 mouse-derived B cells suppressed tumor growth (P<0.05; Fig. 3B).

### JSI124-treated normal B cells inhibit 4T1 tumor growth

**Figure 4:**
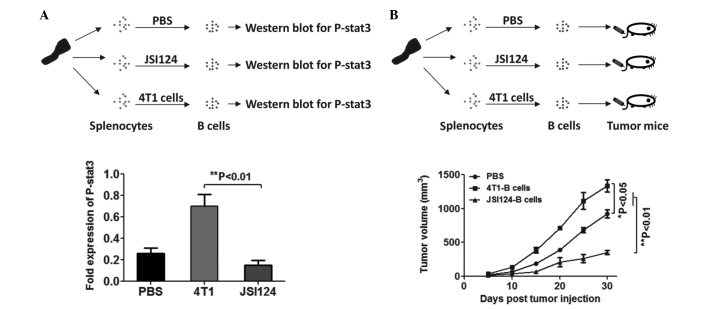
STAT3 expression in untreated B cells or those treated with JSI124, and the analysis of tumor function inhibition. (A) Normal mouse-derived B cells were treated with JSI124 or co-cultured with 4T1 cells, and STAT3 expression was analyzed using western blot analysis. (B) B cells were injected into 4T1-bearing mice and tumor growth was monitored. STAT, signal transducer and activator of transcription; P-, phosphorylated; PBS, phosphate-buffered saline.

To further confirm the tumor inhibitory effect of JSI124-treated B cells, B cells from normal Balb/c mice were treated with JSI124 or co-cultured with 4T1 cells, then the B cells were purified and intravenously injected into 4T1-bearing mice. As shown in Fig. 4A, STAT3 expression was observed to be upregulated in the B cells following co-culture with 4T1 cells, and downregulated in B cells following treatment with JSI124. An *in vivo* tumor therapeutic model revealed that JSI124-treated B cells *in vitro* have a tumor suppressor function *in vivo* (Fig. 4B).

## Discussion

The STAT proteins are a family of transcription factors that consist of seven different members that have roles in normal cellular events, including differentiation, apoptosis, proliferation and the regulation of hematopoietic cell function (15).

The transcription factors of the STAT family are activated by Janus kinase, and the downregulation of this pathway is frequently observed in primary tumors and leads to increased angiogenesis, enhanced tumor cell survival and immunosuppression. Specifically, activated STAT3 promotes tumor cell proliferation (16), survival and invasion (17), and inhibits antitumor immune responses.

Previous studies have shown that STAT3 is a key regulator of tumor growth, metastasis and tumor-associated immunosuppression in patients with malignancies such as breast cancer. More than half of all primary breast tumors and tumor-derived cell lines express constitutively activated STAT3 (18,19). Furthermore, high levels of STAT3 are a poor survival predicator in patients with breast cancer with lymph node metastasis (20). A number of studies have demonstrated that STAT3 inhibitors, including cepharanthine (21), niclosamide (22), cryptotanshinone (23) and JSI124, suppress breast tumor growth. As a STAT3 inhibitor, JSI124 has been extensively used for the treatment of various types of tumor cells, including those of breast cancer. Blaskovich *et al* (2) showed that JSI124 strongly inhibited the growth of MDA-MB-468 human carcinoma cells through targeting the Janus kinase/signal transducer and activator of transcription 3 signaling pathway (2). Lopez-Haber and Kazanietz (9) found that JSI124 also suppresses breast cancer cells by inhibiting Rac1 activation through a reactive oxygen species-mediated and Janus tyrosine kinase 2- and P-Rex1-independent mechanism. However, there have been no studies with regard to the correlation between JSI124 and breast cancer-associated B cells. Olkhanud *et al* (24) reported that B cells evoked by tumors are able to promote breast cancer metastasis by the conversion of resting CD4^+^ T cells into T-regulatory cells. The present study focused on the mechanism of the effect of JSI124 on 4T1 tumor growth. The present study showed that JSI124 suppresses mouse breast 4T1 tumor growth. However, in contrast to these studies, B cells were used as the target of JSI124. In the present study, STAT3 expression in tumor associated-B cells was found to be significantly inhibited by JSI124. Based on a previous study that reported that B cells promote tumor angiogenesis in a STAT3-dependent manner (13), the present study investigated the function of B cells in 4T1 mice, JSI124-treated 4T1 mice, 4T1-cocultured B cells and JSI124-treated B cells *in vitro*. In accordance with the findings of Yang *et al* (13), in the present study, 4T1 tumor-associated B cells were observed to accelerate tumor growth in a STAT3-dependent manner and this acceleration was inhibited by JSI124. In conclusion, the present study has provided a novel tumor suppressor mechanism of JSI124 for the inhibition of mouse breast cancer.
